# Leptin Replacement Improves Cognitive Development

**DOI:** 10.1371/journal.pone.0003098

**Published:** 2008-08-29

**Authors:** Gilberto J. Paz-Filho, Talin Babikian, Robert Asarnow, Karin Esposito, Halil K. Erol, Ma-Li Wong, Julio Licinio

**Affiliations:** 1 Department of Psychiatry and Behavioral Sciences, Center for Pharmacogenomics, University of Miami Miller School of Medicine, Miami, Florida, United States of America; 2 SEMPR – Serviço de Endocrinologia e Metabologia da UFPR, Curitiba, Parana, Brazil; 3 Department of Psychiatry and Biobehavioral Sciences, David Geffen School of Medicine at University of California Los Angeles, Los Angeles, California, United States of America; James Cook University, Australia

## Abstract

**Background:**

Leptin changes brain structure, neuron excitability and synaptic plasticity. It also regulates the development and function of feeding circuits. However, the effects of leptin on neurocognitive development are unknown.

**Objective:**

To evaluate the effect of leptin on neurocognitive development.

**Methodology:**

A 5-year-old boy with a nonconservative missense leptin gene mutation (Cys-to-Thr in codon 105) was treated with recombinant methionyl human leptin (r-metHuLeptin) at physiologic replacement doses of 0.03 mg/kg/day. Cognitive development was assessed using the Differential Ability Scales (DAS), a measure of general verbal and nonverbal functioning; and selected subtests from the NEPSY, a measure of neuropsychological functioning in children.

**Principal Findings:**

Prior to treatment, the patient was morbidly obese, hypertensive, dyslipidemic, and hyperinsulinemic. Baseline neurocognitive tests revealed slower than expected rates of development (developmental age lower than chronological age) in a majority of the areas assessed. After two years, substantial increases in the rates of development in most neurocognitive domains were apparent, with some skills at or exceeding expectations based on chronological age. We also observed marked weight loss and resolution of hypertension, dyslipidemia and hyperinsulinemia.

**Conclusions:**

We concluded that replacement with r-metHuLeptin is associated with weight loss and changes in rates of development in many neurocognitive domains, which lends support to the hypothesis that, in addition to its role in metabolism, leptin may have a cognitive enhancing role in the developing central nervous system.

**Trial Registration:**

ClinicalTrials.gov NCT00659828

## Introduction

Leptin, the product of the *ob* gene, is an adipocyte-derived hormone with multiple functions in reproduction [1], [2], glucose homeostasis [3], [4], bone formation [5], [6], tissue remodeling [7], and inflammation [8], as well as in other elements of the endocrine [9], [10] and immune systems [11], [12]. The most important function of leptin is the regulation of energy expenditure and food intake, due to its actions on the arcuate nucleus of the hypothalamus. In this area, leptin binds to its respective receptors, which are expressed in two different neuronal populations: those that express agouti-related peptide (AgRP) and neuropeptide Y (NPY), and those that express the peptide cocaine and amphetamine-related transcript (CART) and the large precursor peptide pro-opiomelanocortin (POMC). Leptin exerts anorexigenic effects by inhibiting the AgRP/NPY neurons and by stimulating the POMC/CART neurons. Among mice that are leptin-deficient (*ob/ob*) or leptin-resistant (*db/db*), hyperphagia is a constant and obesity, a hallmark.

The effects of leptin on body composition, bone metabolism and on immune, endocrine and metabolic parameters have already been well documented. It has also been shown that treatment with leptin in its synthetic form (recombinant methionyl human leptin; r-metHuLeptin) leads to structural [13] and functional changes within specific regions of the central nervous system [14], [15]. Other roles of leptin within the brain include effects on neuron excitability and on synaptic plasticity [16]–[18]. In addition, leptin maintains neural stem and progenitor cells, affects neural differentiation, promotes the migration of neuronal lineage cells to the cortical plate, and regulates the development of hypothalamic feeding circuits [19]–[21]. Taking all these actions together, it is feasible to hypothesize that leptin is also important for the development of neurocognition. There is no data in the literature addressing the effects of leptin on the development of neurocognition in humans. The evaluation of these effects in normal humans has many limitations, such as the ethical problems in administering exogenous leptin to normal children and the fact that the child's endogenous leptin is an evident confounding factor.

Our group has previously described the phenotypic findings in the only leptin-deficient patients identified at adulthood to date. Concomitantly, we described the effects of leptin-replacement therapy in these unique patients [13], [14], [22]–[25]. These patients are two women and one man from a highly consanguineous extended Turkish pedigree, who carry a nonconservative missense leptin gene mutation (Cys-to-Thr in codon 105). These patients have benefited from a daily injection of recombinant methionyl human leptin (r-metHuLeptin) and have shown significant improvements regarding body weight, gonadal function and behavior. To our knowledge, 10 children with congenital leptin deficiency, also due to loss-of-function mutations in the gene encoding leptin, have been identified in the world [26]–[30].

Here we present a unique human model that overcomes the limitations of administering exogenous leptin to normal children, in order to evaluate its effects on cognition. We show, for the first time, the effects of replacement with r-metHuLeptin on neurocognition in a leptin-deficient boy from the aforementioned consanguineous Turkish family. Additional data on the anthropometric and metabolic parameters are described, before and during treatment.

## Methods

The protocol for this n-of-1 trial and supporting CONSORT checklist are available as supporting information; see Protocol S1 and Checklist S1.

### Patient

The patient is a 7-year-old male born to a highly consanguineous Turkish family. His parents are first-degree cousins. Due to excessive weight gain beginning at age 3 months, and to familial history of leptin deficiency caused by a mutation in the leptin gene, that gene was genotyped when he was 2 years old.

### Genotyping

The nucleotide sequence of the *ob* gene was analyzed in the proband and his first-degree relatives by direct sequencing. The patient was homozygous for a missense leptin gene mutation (Cys-to-Thr in codon 105), whereas the parents and the sister were heterozygous for the same mutation.

### Leptin administration and dose

Leptin replacement therapy was started at age 5y1m. Recombinant methionyl human leptin (r-metHuLeptin) was supplied initially by Amgen, Inc. (Thousand Oaks, CA, USA), and subsequently by Amylin Pharmaceuticals, Inc., (San Diego, CA, USA). The drug has been uninterruptedly administered once a day in the evening (18:00–20:00), with a starting dose of 1.36 mg/day SQ. Dosage was initially calculated to achieve a peak serum leptin concentration of 70 ng/ml, which is equivalent to 10% of the child's predicted normal serum leptin concentration (based on age, sex, and body composition) [31], [32]. Due to his decreased food intake and subsequent weight loss, dosage was progressively reduced to 0.45 mg/day, which is the current dose, at age 7y5m. We chose daily evening administration to model the normal circadian variation of endogenous leptin, which is characterized by a pulsatile circadian rhythm with a marked nocturnal rise [33].

### Neurocognitive evaluations

The patient underwent a neurocognitive evaluation pre-treatment (T1, at age 5y1m) and twice following the initiation of leptin treatment (T2, at age 6y0m; T3, at age 7y2m). A fluent Turkish-speaking clinician who was familiar with all test items translated the test instructions and language based measures. The clinician was familiar to the patient and had established good rapport. The patient generally showed interest in the tasks and readily participated but showed inconsistent attention. In order to maintain cooperation and interest in the test items, M&M candies were offered as needed to the patient at the T1 and T2 test sessions but were not necessary at T3.

The following neurocognitive assessments were administered: 1) Differential Ability Scales (DAS), a measure of general verbal and nonverbal functioning; and 2) selected subtests from the NEPSY, a measure of neuropsychological functioning in children. Both measures are standardized, commercially available instruments that are used for clinical and research purposes. Although both the DAS and the NEPSY are normed on a representative sample based on the US population census and on English speaking children only, careful consideration was given to selecting instruments (and subtests from these instruments) that tap into general skills and are least likely to be affected by cultural factors. We are fully aware that language and cultural differences may have introduced biases to the test results. Nonetheless, the longitudinal design was a strength in the study as it allowed us to use within subject analyses (with the patient's pre-treatment performance serving as his own control) to describe changes in cognitive functioning after the initiation of leptin treatment. Therefore, any aspects of culture or language status that may have affected the test results would have a similar effect on the patient's performance at all three time points and therefore cancel out in the analyses.

We assumed that the level of cognitive functioning at the pre-treatment evaluation was representative of the patient's cognitive functioning from birth until the baseline evaluation. This assumption seemed reasonable given that the effect of leptin on brain development likely began in utero. Rates of development were computed for each of the three sets of time points: T1) from birth to 61 months (pre-treatment), T2) from 61 months to 72 months (one year post-treatment), and T3) from 72 months to 86 months (two years post-treatment). To assess changes in cognitive functioning pre- and post-treatment, age equivalencies (based on typical performance for a given age or age range) were derived using normative data from the test manuals. For example, at age 4, performance at the level of a 4-year-old would yield a rate of development of 1 (4/4). Therefore, a ratio of “1” indicates typical development. In contrast, at age 4, a performance at the level of a 3-year-old would yield a developmental rate of 0.75 (3/4). Subsequently, a score of <1 indicates performance (developmental age) below that expected for chronological age. Rates above “1” indicate development above expected levels.

In addition to the cognitive measures, parent report measures assessing behavioral/emotional problems (Achenbach Child Behavior Checklist, CBCL) and behavioral regulation/executive functioning (Behavioral Rating Inventory of Executive Function, BRIEF) were orally translated and administered to the patient's mother.

### Hormonal and metabolite assays

Blood samples were obtained after overnight fasting and analyzed for TSH, free T4, T3, cholesterol, triglycerides, HDL cholesterol, LDL cholesterol, glucose, and insulin, using standard assays, at T1 and T3.

### Food intake

At T1 and T2, the General Clinical Research Center at the University of California, Los Angeles (UCLA GCRC) provided food *ad libitum*. During these visits, intake was calculated by subtracting content of uneaten food and beverage items from initial content of each item served. All food and beverages provided to the patient were recorded by weight (in grams, to nearest 0.1 g) with a precision balance (Mettler-Toledo Inc., Columbus, OH). The patient's mother was also instructed to keep dietary records, which were carefully reviewed by the GCRC Senior Dietician. Nutrient intake was calculated using a nutrition analysis software (Nutritionist Pro, First DataBank, Inc., San Bruno, CA). At T3, meals were not provided by the GCRC, as the participant was evaluated as an outpatient. Nevertheless, he was allowed to eat *ad libitum*, and dietary intake was assessed through food records kept by his mother. The software Food Processor SQL (Esha Research, Salem, OR, USA) was used for nutritional analysis in 2007.

### Research ethics

At T1 and T2, the study took place at the General Clinical Research Center at the University of California, Los Angeles (UCLA GCRC). The study protocol was approved by the Food and Drug Administration and by the UCLA Institutional Review Boards. At T3, the study site was relocated to the University of Miami, where new approvals from the local Institutional Review Boards were obtained. Informed parental consent/patient assent (at age 7) were obtained for all studies. Written informed consent for publication was given by the mother.

## Results

### Neurocognitive development

**Figure 1:**
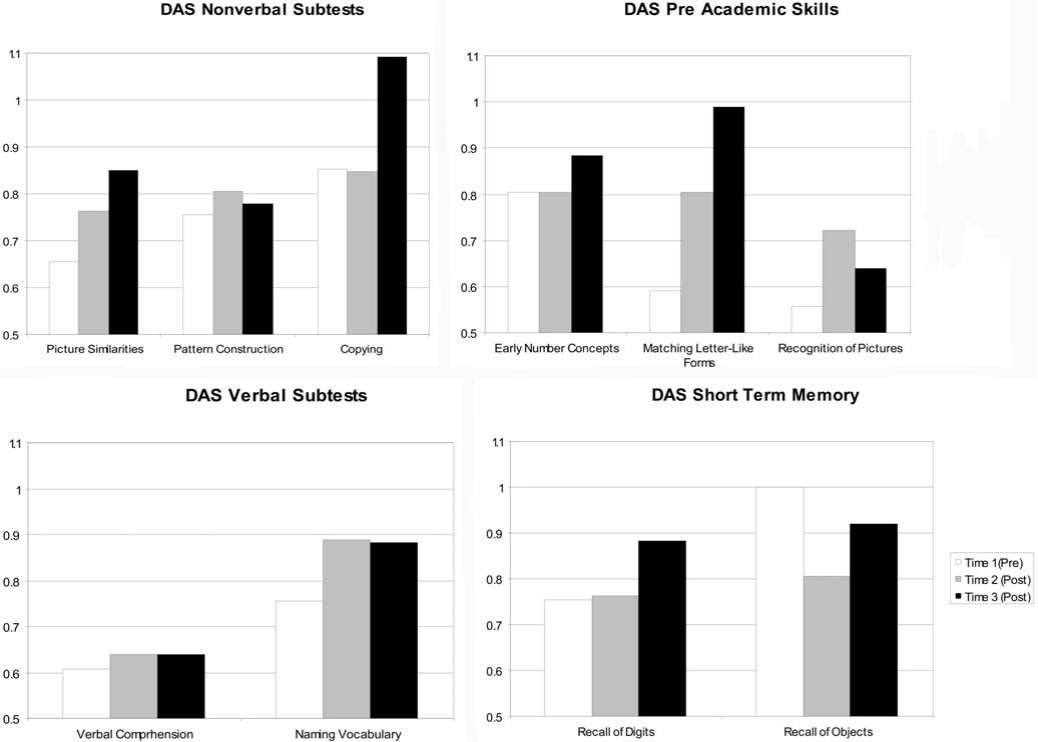
Rates of development for DAS subtests. A rate of “1” (vertical axis) indicates typical development (i.e., developmental age equals chronological age). For example, at age 4, performance at the level of a 4 year old would yield a rate of development of 1 (4/4). In contrast, at age 4, a performance at the level of a 3 year old would yield a developmental rate of 0.75 (3/4). Subsequently, a score of <1 indicates performance (developmental age) below that expected for chronological age.

General cognitive ability was assessed by the DAS, which measures verbal, nonverbal, short-term memory, and pre-academic skills. The patient's pre- and post- treatment DAS verbal and nonverbal cluster scores were lower than the scores for age- matched controls from the normative sample. However, administration of r-metHuLeptin was followed by an upward trend in development, with scores generally normalizing by T3. Pre-treatment verbal and nonverbal scores were in the 4^th^ percentile. At T2, verbal scores were in the 9^th^ percentile while nonverbal scores were in the 6^th^ percentile. In contrast, by T3, the patient's verbal scores were in the 14^th^ percentile while his nonverbal scores were in the 30^th^ percentile, both broadly within normal limits for his age. With one exception (“Recall of Objects”), Figure 1 reveals that the patient's pre-treatment rate of development was consistently lower than expected for all DAS subtests administered. Of note however, the patient's rate of development increased, especially by T3, and in several instances achieved levels generally within the expected range for his age (i.e., close to “1”).

**Figure 2:**
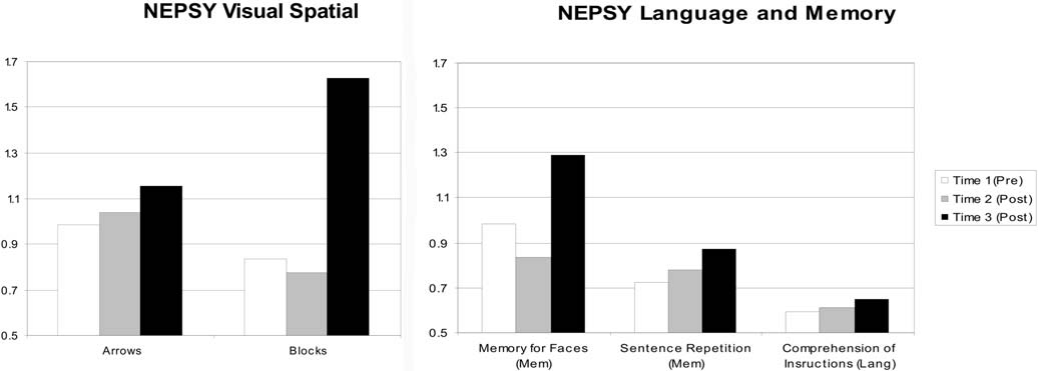
Rates of development for NEPSY subtests. A rate of “1” (vertical axis) indicates typical development (i.e., developmental age matches chronological age). A score of <1 indicates performance (developmental age) below that expected for chronological age.

Neuropsychological functioning was evaluated by the NEPSY, with results analyzed similarly to those of the DAS (described in Methods). Similar to his cognitive results, the patient's intra-treatment rate of development showed an increasing trend compared to his pre-treatment rate of development, with many T3 rates of development at or exceeding age expectations (e.g., Memory for Faces, Blocks). The NEPSY scores are illustrated in Figure 2.

Parental report of emotional problems and behavioral regulation resulted in scores that were within normal limits compared to age-matched peers on all three occasions. The patient's mother did not report any concerns except for his overall health and well-being.

### Metabolic and anthropometric parameters

**Figure 3:**
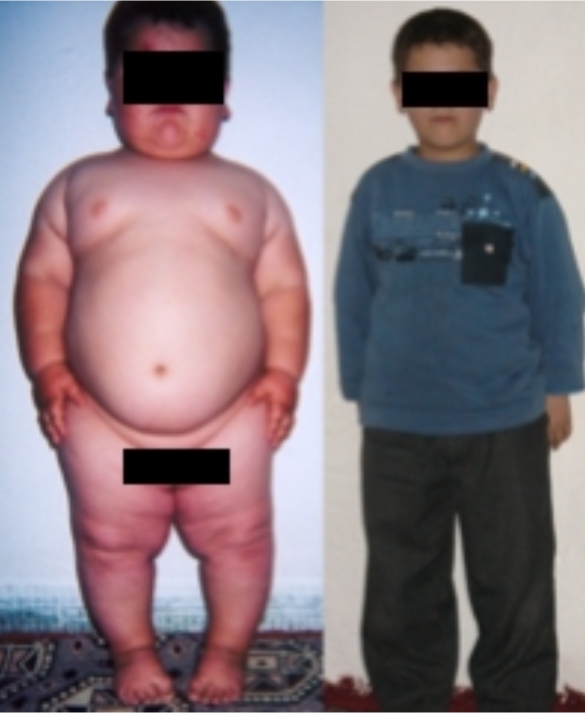
Patient before replacement with r-metHuLeptin at age 5y1m and during treatment at age 7y2m.

Ten months after initiation of therapy, his growth velocity was normal (5.7 cm/year), but it decreased slightly to 4.1 cm/year, when calculated between ages 5y11m and 7y2m. Figure 3 and Table 1 illustrate the changes in anthropometric parameters.

**Table 1:** Weight, height and BMI before and after treatment initiation, correlated with percentiles for same sex and age.

	5y1m	5y11m	7y2m
	Before treatment initiation (T1)	10 months after treatment initiation (T2)	25 months after treatment initiation (T3)
Weight (percentile for age)	47.0 kg (>97^th^ percentile)	32.3 kg (>97^th^ percentile)	33.7 kg (97^th^ percentile)
Height (percentile for age)	109 cm (50^th^ percentile)	114 cm (50^th^ percentile)	119 cm (25^th^ percentile)
BMI (percentile for age)	39.6 kg/m^2^ (>97^th^ percentile)	24.8 kg/m^2^ (>97^th^ percentile)	23.8 kg/m^2^ (>97^th^ percentile)

On baseline clinical examination, the patient's blood pressure measured 110/70, slightly above the 90^th^ percentile for his age [34]. At age 7y2m, his blood pressure was normal, at 101/66 mmHg. Other vital signs were normal at all times and, besides obesity, there were no other abnormalities. At baseline, the patient had normal liver and renal functions and was euglycemic at all times, but presented with hyperinsulinemia before treatment. This was normalized as his weight started to decrease. Also, there were decreases in triglycerides, total cholesterol and LDL cholesterol, and an increase in HDL cholesterol. Thyroid function was normal at all times (Table 2).

**Table 2:** Lipid profile, glucose and insulin before and after treatment initiation.

	T1	T3
Glucose (mg/dl)	79	87
Insulin (uU/ml)	21	7
Cholesterol (mg/dl)	166	155
LDL (mg/dl)	87	66
HDL (mg/dl)	36	65
Triglycerides (mg/dl)	216	120
TSH (mU/l)	3.8	4.57
Free T4 (ng/dl)	1.3	1.2
T3 (ng/dl)	154	167

### Caloric intake

Before treatment, average caloric intake was 2,709±370 kcal/day (54.3±2.5% from carbohydrate, 32.6±1.6% from fat and 13.1±1.1% from protein). In the 4^th^ week of treatment, average caloric intake decreased to 1,751±475 kcal/day (56.0±5.9% from carbohydrate, 32.6±2.9% from fat and 11.5±3.2% from protein). One year after treatment initiation, average daily caloric intake was 2,594±346 kcal (55.8±5.6% from carbohydrate, 31.0±3.5% from fat and 13.2±2.8% from protein). Two years after treatment initiation, average daily caloric intake was 2,194±292 kcal (64.5±3.12% from carbohydrate, 16.6±3.0% from fat and 15.1±5.16% from protein), which is 106% of the recommended caloric intake for a boy this age, height and weight.

## Discussion

In this n-of-1 trial, we observed that leptin was associated with an improved rate of development in various aspects of neurocognitive functioning. As evidence that leptin replacement was beneficial to the patient, we documented resolution of hypertension, dyslipidemia and hyperinsulinemia, in the context of decrease in food intake and weight loss. To the best of our knowledge, this is the first longitudinal study that shows that leptin has a cognitive enhancing role in the developing CNS of humans. To achieve that result, we employed the only possible human model: a leptin-deficient child under leptin replacement therapy. Treatment of leptin-sufficient children poses many ethical issues, and results are contaminated by the fact that these children are already under the effects of their endogenous leptin. Due to the leptin-naïve status of the patient, we were able to evaluate the effects of leptin on the developing CNS.

To compute rates of development at different time points, we inferred a steady rate from birth to 61 months (pre-leptin treatment) because we did not have two pretreatment baselines. The inferred rate of development was much slower from birth to 61 months (T1), suggesting developmental age below expectations for chronological age, than the rate of development observed during the treatment phase (T1 to T3) on most of the neurocognitive measures administered. By T3 (two years after initiation of leptin therapy), developmental relative chronological age ratios increased on all but one subtest compared to the baseline evaluation, and in many cases, approached or exceeded chronological age expectations. Because the patient had not experienced any environmental changes that would affect his neurocognitive abilities (i.e., starting school, receiving tutoring) between T1 and T2, we cautiously attribute the increased rate of development to leptin treatment and its subsequent effects on the CNS. However, the patient had completed the first year of school in his home country by the time of the third evaluation, which may partly have contributed to the apparent increase in rate of development between T2-T3. Nonetheless, these improvements are consistent with the finding that leptin replacement tends to normalize certain brain structures in leptin deficient mice. It would be instructive to compute correlations between rates of change in cognitive scores and rates of change in brain volume pre- and post-treatment to further support this assertion. Nonetheless, initial results suggest that in this leptin deficient patient, leptin may have had a cognitive enhancing role.

After the initiation of r-metHuLeptin, we observed a slower rate of development at T2 relative to T1 on a few subtests (“Recall of Objects,” “Memory for Faces,” and “Blocks”). However, scores at T2 were no more than 20% lower than scores predicted for age and in most cases, not clinically different. The few exceptions to the general pattern of increase in developmental rate may in part be within the error variance of the instrument. These normal variations in performance are difficult to characterize given that there is only subject in the study.

Leptin has a role in the central nervous system beyond its regulatory function through the hypothalamus on food intake and energy balance. Leptin is expressed in the brain, including the cerebellum, pyriform cortex, cerebral cortex, thalamus, hippocampus, amygdala, olfactory tract and substantia nigra [35]. It may improve cognition by the selective enhancement of N-methyl D-aspartate (NMDA) receptors, facilitating long-term potentiation (LTP) [36]. In low frequency stimulation, leptin inhibits hippocampal neurons, regulating hippocampal excitability [35], [37]. In adult mice, leptin increases hippocampal neurogenesis, both in vitro and in vivo [38]. Hippocampal synaptic plasticity is also regulated by leptin. Animals with leptin receptor mutations (*db/db* mice or *fa/fa* rats) present deficits in hippocampal-specific memory tasks [39], impairments in hippocampal LTP, and long-term depression [36]. Leptin is also an important hormonal signal in the developing CNS. In adult leptin deficient (*ob/ob*) or leptin resistant (*db/db*) mice, for example, leptin deficiency leads to lower brain weight and protein content [21], [40], [41]. Many of these effects normalize after postnatal administration of leptin [35]. In addition, brain myelin [42], neuronal soma size [43], and several synaptic proteins are reduced [35]. Growth-associated proteins in the neocortex, striatum and hippocampus are elevated, and dendritic orientation is altered [44].

In humans, normal neonates have two to three times higher serum leptin levels, when compared to adults [45]. Elevated circulating leptin levels are originated from the maternal and the neonatal adipose tissue, as well as from the placenta. Moreover, the increased adipose leptin mRNA expression in fetal adipose tissue, and the delayed onset of synthesis of leptin prior to the maturation of its receptor also explain the high levels of leptin [46]. Several maternal, placental and neonatal factors are potentially associated with production and functions of leptin in early life, and have been reviewed by Alexe et al. [47]. Leptin does not appear to regulate food intake and body weight during neonatal life [48], and the metabolically irrelevant surge of leptin may act as a developmental signal [49], which coincides with the development of major hypothalamic feeding circuits [50]. This neurodevelopmental activity of leptin is limited to a neonatal window of maximum sensitivity, corresponding to a period of elevated leptin secretion [51]. However, in adults, leptin can still affect brain plasticity by causing synaptic rearrangement of excitatory and inhibitory inputs on arcuate neurons [18], suggesting that these circuits remain relatively plastic throughout life.

In abnormal conditions, low levels of leptin at birth are seen in small for gestational age infants [52], but the neurodevelopmental consequences of this alteration have not been evaluated. The neurodevelopmental actions of leptin are not restricted to the hypothalamus: it has been demonstrated that cortical and hippocampal development can also be influenced by leptin [53], [54]. It is known that, besides its effects on the components of feeding circuits, leptin also acts as a cognitive enhancer in the hippocampus and on excitatory synaptic strength, which may regulate the processes involved in learning and memory 17, 36. These processes would be explained, at the cellular level, by the leptin-mediated modulation of hippocampal synaptic plasticity, leading to the conversion of hippocampal short-term potentiation (STP) into LTP, via enhancing NMDA receptor function [55]. In the hippocampus, leptin also regulates neuronal excitability via its ability to activate the large conductance Ca^2+^-activated K^+^ (BK) channels. Leptin can also evoke a novel form of NMDA receptor-dependent LTD in the CA1 region of the hippocampus, but this is only apparent under conditions of enhanced excitability [56], [57]. The signaling processes underlying these effects involve activation of a phosphatidylinositol 3-kinase-dependent process [35]. Activation of mitogen-activated protein kinases and Src tyrosine kinases has also been implicated in this pathway.

Morphological studies in adult humans with genetic leptin deficiency suggest that leptin effect on the CNS extends beyond the hypothalamic nuclei. Six months of leptin replacement treatment for these three genetically leptin-deficient adults produced increased gray matter concentration in the anterior cingulate gyrus, inferior parietal lobule, and the cerebellum, findings that were maintained for 18 months following treatment [13].

The most marked effect of leptin replacement is weight loss, which is primarily achieved through reducing caloric intake. Leptin also leads to weight loss by increasing resting energy expenditure (REE) in animals [58], but not in humans [29]. In this study, we did not evaluate the effects of leptin on REE, which prevents us from stating that weight loss was entirely attributed to the decrease in energy intake. Following weight loss, we observed a mild deceleration in growth. Height changed from the 50^th^ percentile to the 25^th^ percentile between the first and the second year under treatment. Growth will be monitored and adequate hormonal parameters will be assessed in the next follow-up visit. Subsequent to losing weight, hypertension, dyslipidemia and hyperinsulinemia were reversed. We believe these changes are attributable to weight loss, but we cannot exclude a direct effect of leptin on these parameters, independent of weight loss. In particular, it is known that leptin directly affects the pancreatic beta cells to decrease insulin secretion [59], but it is problematic to evaluate whether these changes are caused by leptin alone, by weight loss or both.

We acknowledge that our study has some limitations. First, the significance of our findings is hard to judge, due to the fact that we report the findings on only one patient. To our knowledge, eleven leptin-deficient children have been identified in the world thus far (including our patient). Neurocognitive evaluations of these children are warranted, preferably at early ages, and while leptin-naïve. Second, we did not have an age-, sex-, weight- and cultural background-matched control group. To compensate for this limitation, we gave careful consideration to our choice of measurement instruments so as to select tests and subtests that are less culturally biased and measure general skills. Further, we considered a strength of the study to the fact that we have longitudinal evaluation, which in essence allowed us to use the patient's baseline performance as his own control for subsequent evaluations. Although this is an n-of-1 trial, we chose not to conduct additional leptin-free evaluations (other than those performed at T1) because we believe that the absence of leptin would be harmful to the child's development.

In conclusion, treatment of a leptin-deficient child with r-metHuLeptin was beneficial, leading to improvements in neurocognition, substantial weight loss, decrease in caloric intake and normalization of the parameters of metabolic syndrome. Further studies need to confirm these neurocognitive findings additional in leptin-deficient patients across the life span. Additional findings may support a rationale for the development of further studies to test the hypothesis that the effects of leptin replacement may improve cognition in leptin-sufficient patients with delay or decline of cognitive function.

## Supporting Information

Protocol S1 Trial Protocol.
(0.80 MB DOC)

Checklist S1 CONSORT Checklist.
(0.06 MB DOC)
