# Correction: Leptin Replacement Improves Cognitive Development

**DOI:** 10.1371/annotation/da7a6fa5-77f1-4bd8-aadf-b7fb8859ea8b

**Published:** 2008-10-29

**Authors:** Gilberto J. Paz-Filho, Talin Babikian, Robert Asarnow, Tuncay Delibasi, Karin Esposito, Halil K. Erol, Ma-Li Wong, Julio Licinio

Because the author Tuncay Delibasi was added to the author byline after publication, the Author Contributions section does not correctly reflect the new authorship. The new Author Contributions should read:

Conceived and designed the experiments: GPF TB RA TD KE MLW JL. Performed the experiments: GPF TB RA TD KE HKE MLW JL. Analyzed the data: GPF TB RA TD KE MLW JL. Contributed reagents/materials/analysis tools: RA MLW JL. Wrote the paper: GPF TB RA TD KE MLW JL.

