# Correction: Leptin Replacement Improves Cognitive Development

**DOI:** 10.1371/annotation/df013c27-a849-4ce6-990b-e6cad0d95fea

**Published:** 2008-10-29

**Authors:** Gilberto J. Paz-Filho, Talin Babikian, Robert Asarnow, Tuncay Delibasi, Karin Esposito, Halil K. Erol, Ma-Li Wong, Julio Licinio

Dr. Tuncay Delibasi was not included in the author byline. He should be listed as the fourth author. This also affects the article's citation. Please view the corrected author byline, affiliations, and citation here:

Gilberto J. Paz-Filho^1,2^, Talin Babikian^3^, Robert Asarnow^3^, Tuncay Delibasi^4^, Karin Esposito^1^, Halil K. Erol^1^, Ma-Li Wong^1^, Julio Licinio^1*^

**1** Department of Psychiatry and Behavioral Sciences, Center for Pharmacogenomics, University of Miami Miller School of Medicine, Miami, Florida, United States of America; **2** SEMPR – Serviço de Endocrinologia e Metabologia da UFPR, Curitiba, Parana, Brazil; **3** Department of Psychiatry and Biobehavioral Sciences, David Geffen School of Medicine at University of California Los Angeles, Los Angeles, California, United States of America; **4** Department of Endocrinology and Metabolism, Ankara Numune Education and Research Hospital, Ankara, Turkey

* E-mail: jlicinio@med.miami.edu

**Citation:** Paz-Filho GJ, Babikian T, Asarnow R, Delibasi T, Esposito K, et al. (2008) Leptin Replacement Improves Cognitive Development. PLoS ONE 3(8): e3098. doi:10.1371/journal.pone.0003098

