# Supplementary material for: Leptin Replacement Improves Cognitive Development
Source: PLoS One. 2008 Aug 29;3(8):e3098. doi: 10.1371/journal.pone.0003098 (PMC2518120; doi:10.1371/journal.pone.0003098)
Supplement: Protocol S1 — Trial Protocol. (0.80 MB DOC) [file pone.0003098.s001.doc]

| **University of Miami ▪ Human Subjects Research Office**  JMTE 1500 NW 12^th^ Avenue ▪ Ste. 1000 ▪ Miami, FL 33136 ▪ Tel.: 305-243-3195 ▪ Web: [**https://hsro.med.miami.edu**](http://hsro.med.miami.edu/) | 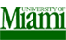 |
| --- | --- |
| **Form HSR-1G: New Research Protocol Form – Detailed Protocol Information** |

**Instructions: This form is mandatory for all new protocol submissions and must accompany Form HSR-1. Please complete all information requested; for those questions that are not applicable, please state “not applicable” or “N/A” as your response. The form must be typed; handwritten or incomplete forms will not be processed.**

| **Principal Investigator:** | | | |
| --- | --- | --- | --- |
| **Name:**  Julio Licinio | **Title:**  MD | | **Employer:**  **[X] UM** **[ ]** **JHS** |
| **Department:**  Dep Psychiatry and Behav Sciences | **Division:** | | **Mail Code:** |
| **Email Address:**  jlicinio@med.miami.edu | **Telephone:**  (305) 355-9105 | **Fax:**  (305) 355-9099 | **Beeper:** |

**Research Protocol Title:**

Effects of Leptin Replacement in Children

**12.1 Research Background:**

12.1.1 Please provide a brief summary of the present knowledge relevant to the research, and make citations to applicable scientific literature. Explain how the research may contribute to the advancement of knowledge:

We will assess the endocrine and immune effects of leptin replacement in leptin-deficient children, from a consanguineous Turkish family. In a separate protocol (IRB # 01-01-019-03) we have studied adult members of this family. We now propose to study the effects of leptin replacement in children who are deficient in leptin due to a genetic defect. We hypothesize that leptin replacement will have significant effects on immune and endocrine function.

**Background:**

Childhood obesity is becoming an epidemic worldwide. In the 10 years between the National Health and Nutrition Examination Survey (NHANES) II (1976-1980) and NHANES III (1988-1991) the prevalence of overweight in the US, based on body mass index corrected for age and sex, has increased by approximately 40% (to 11% in the 6-11 year age group)(1). The immediate adverse effects of childhood obesity include orthopedic complications, sleep apnea and psychosocial disorders. As obese children are more likely to become obese adults, we may expect to see profound public health consequences as a result of the emergence in later life of associated co-morbidities such as type 2 diabetes and cardiovascular disease (2).

Results of research have provided new insights into the physiological basis of bodyweight regulation. Leptin, an 16-kDa adipocyte-derived hormone, has been implicated in the regulation of food intake and metabolism(3). Leptin is synthesized in humans primarily but not exclusively by adipose tissues (4). Its concentration in circulating plasma is usually proportional to body fat mass, but is higher in women than in men because of a combination of factors that include not only different distribution of and greater fat mass in women, but also the fact that testosterone reduces its level in men (5). Even though leptin has striking effects in animal models, its role in human biology is less clear. In a recent review, Himms-Hagen points out that the biology of leptin in the mouse is different from humans, because the metabolism of the mouse is very distinct from that of humans in many respects, particularly in the far more drastic ways it conserves energy when it has to very rapidly adapt to lack of food. These include not only suppression of reproduction, but also lowering of body temperature (torpor), suppressing thyroid function and growth, and increasing secretion of stress hormones (from the adrenal). Thus, several observations on actions of leptin in mice are not applicable to humans (6). Clinical investigation is therefore necessary for the understanding of the biology of leptin in humans. While studies of humans with an intact leptin molecule can be informative, the opportunity to replace leptin in leptin-naive subjects and simultaneously study the effects of leptin replacement on endocrine function would give us a unique opportunity to understand the endocrine effects of human leptin.

Recently, human congenital leptin deficiency, a single gene disorder resulting in early onset obesity has been characterized(7). Montague *et al.* reported that two children homozygous for a frameshift mutation in the leptin gene (deletion of a glycine at residue 133) which results in a truncated protein (7). These were two cousins within a highly consanguineous family of Pakistani origin with severe obesity of early onset: an 8 year old girl weighing 86 kg, and a 2 year old boy weighing 29 kg. These children were noted to be severely hyperphagic, constantly demanding food, with an intense drive to eat. One year of recombinant leptin replacement therapy resulted in marked improvements in hyperphagia and body fat mass in the older child (8).

In an extended Turkish family, Strobel *et al.* have identified 4 homozygous and more than 8 adult individuals heterozygous for a functional mutation of the leptin gene(9). This mutation is identical to the mutation, which leads to the appearance of a premature stop codon in the leptin gene of the *ob/ob* mouse. COS-1 cells transfected with the intact leptin molecule secrete leptin into the culture medium; in contrast in COS-1 cells transfected with a mutant construct leptin can be recovered in the cell lysate, but not in the medium, indicating that the mutant protein is synthesized but not secreted. These results suggest that this mutation does not induce protein breakdown, but rather impairs the normal processing of leptin through the intracellular secretory pathway (9). The adult patients from this family, in addition to morbid obesity had hypogonadism.

We have recently identified a three year old boy with same mutation. In this young patient, a C→T substitution was identified in codon 105 of the leptin gene, resulting in an Arg→Trp replacement in the mature protein in the same family (9).

It is noteworthy that all 19 normal-weight individuals in the youngest generation of this family are alive. In contrast, 7 of 11 obese individuals who had the phenotype that is characteristic of leptin gene mutation (normal weigh at birth, with voracious appetite starting in the neonatal period, and doubling of body weight in the first 4 months of life) died in childhood following infections. The odds ratio for mortality in the context of this obesity phenotype is 25.4 (*P* < 0.01, Yates test), indicating that this mutation severely impairs key biological functions during childhood, with a negative impact on survival (10). Thus, it is clinically indicated to treat the three surviving adults and homozygous three year old boy with recombinant human leptin. Such treatment presents an unparalleled opportunity for research in endocrinology and metabolism, as we will be able to ascertain the effects of leptin replacement in human biology.

We are conducting detailed studies of endocrine function using a rapid-sampling protocol during the course of leptin replacement in three morbidly obese adult members of this family who are the only leptin-naive adults identified in the world so far. Our aim in that project is to evaluate the definitive ascertainment of the effects of leptin on endocrine function in humans. Exogenous leptin treatment of healthy individuals adds leptin to the body, but it is impossible in that case to ascertain whether any of the observed changes are due solely to the effect of exogenous leptin or to the combined effect of exogenous plus highly variable and pulsatile endogenous leptin secretion. Another confounding factor is that the extent to which exogenous leptin administration may affect endogenous leptin output is unknown. Moreover, if exogenous leptin causes small or no changes in endocrine outcome measures in healthy volunteers, it is not possible to determine if that is because leptin has indeed limited or no effect on those parameters, as in that case endogenous leptin levels might have already contributed maximally to the outcome measures. A study of leptin-naive subjects avoids all of these confounding factors and pitfalls, because the only bioactive leptin to which they will be exposed is the exogenously administered recombinant protein.

The results of our ongoing trial of leptin replacement therapy in adult leptin-naïve patients are impressive. The mean BMI of three patients dropped from 51.2 kg/m2 at baseline to 26.9 kg/m^2^ after 18 months of treatment. Parallel to the decrease in weight, mean physical activity level is increased. Leptin replacement resolved morbid obesity, hypogonadism in all patients and one of the patients who had type 2 diabetes have normal blood glucose and HbA1C levels after weight loss.

The proposed study of the treatment of a child with congenital leptin deficiency will permit to elucidate key aspects of human endocrine and immune function, and will give new insights on the role of leptin in human endocrine regulation. We propose here to study this child for an initial period of one-year. If he responds well to the treatment, as we expect based on the data obtained from his adult relatives, we would apply for future extensions of this study. Conversely, if for unexpected reasons, the clinical response is not favorable, it would not be reasonable to extend the study. Therefore, we believe it is justified to propose an initial study period of one year, and to re-assess the overall duration of the study after leptin replacement treatment is initiated and clinical responses are documented.

We have previously demonstrated that leptin appears to affect the dynamics of human endocrine rhythms in adults (5, 11) . In rodents, leptin deficiency is associated with hyperphagia, impaired energy expenditure and blunted fat oxidation. Leptin administration in leptin deficient boy will possibly increase energy and fat metabolism by increasing sympathetic nervous system activity. To test this hypothesis, we will measure food intake, energy expenditure, body composition and sympathetic nervous system activity in 3-year old boy who is homozygous for a leptin gene mutation before and throughout the leptin replacement therapy.

Leptin modulates T-cell function and affects the phagocytic activity of macrophages (12). Seven members of this family who had a phenotype identical to those of our leptin-mutated subjects (-/-) died in infancy and childhood following infections. In contrast, all the non-obese members in the youngest generation of this family are alive; they live in the same area as their obese relatives and have the same socio-economic status and access to medical care (10). Thus, immune function will also be assessed during the course of this study. Specifically, tests for antibody, complement and phagocytic function, tests for T-cell immunity, flow cytometry, TREC PCR, thymus imaging studies will be performed. Antibody levels for pathogen organisms will be checked and the child will be vaccinated if needed.

Because there are preliminary results from these patients supporting the concept that leptin contributes to regulate bone mineral density (10), bone function will also be assessed in this leptin deficient child.

There is very limited knowledge on the consequences of leptin deficiency on bone and mineral metabolism in humans. Several recent reports have indicated an important regulatory function of leptin on bone formation in mice; however such findings are still controversial. Ducy et al. (13) reported that two murine models of obesity, the leptin deficient ob/ob mice and leptin receptor deficient db/db mice have a phenotype associated with increased bone formation rates and high bone mass. The authors demonstrated reversal of the high-bone mass phenotype in ob/ob mice that were leptin-deficient following intracerebroventricular infusions of leptin, although they could not demonstrate leptin receptors on osteoblasts. They concluded that leptin modulates bone mass by a central effect, and suggest leptin deficiency or insensitivity is responsible for the decreased bone mass in these mice (13).

On the other hand, we have reported alterations in PTH-calcium and bone mineral density in leptin-deficient adult patients (10). Male patient had normal PTH levels, while his BMD of spine showed osteopenia. In contrast, female patients had normal BMD. Thus, preliminary data in the adult leptin-deficient patients supports the comment that leptin plays a role in controlling bone mass. More recently, Farooqi et al, (14) reported that BMD and bone mineral content were appropriate for age and gender in leptin-deficient state although skeletal maturation was increased in three leptin-deficient Pakistani children with a different mutation of leptin. But, there are limitations with the assessment of bone mass by DEXA in children (15).

Computed tomography introduced the technique of digital imaging to diagnostic radiology. The transverse anatomic sections afforded by this digital technique provide a three-dimensional image unobscured by overlying structures. The data displayed as the CT image are actually a representation of attenuation values or CT numbers of the object scanned. These numerical values are stored in digital form and are accessible for future study, as needed. The use of this digital information to provide quantitative information has been labeled quantitative computed tomography (QCT).

QCT is an established technique for measuring bone mineral density in the axial and appendicular skeleton (16, 17). The average scanning time is less than 10 minutes, and the examinations are performed using a commercial CT scanner and a bone mineral reference standard to calibrate each scan. The procedures for bone mineral measurements of axial and appendicular bone differ slightly. Based on a lateral localizer image, or scoutview, single 10-mm-thick sections are obtained through the midplane of each vertebra (usually two to three vertebrae of T12-L4) using a low-dose technique, with the gantry angled parallel to the vertebral endplates. A region of interest (ROI) is then positioned in the interior portion of trabecular bone of the vertebral body for analysis (16, 17). In some approaches this ROI may be positioned automatically (18, 19). The CT density of the selected area of interest is measured in Hounsfield units (HU), which is an unit of measurement based on an arbitrary scale of 1,000 positive values ranging from 0 (the value of water) to 1,000 (the attenuation of compact bone) and 1,000 negative values, where –1,000 corresponds to the attenuation coefficient of air. Conversion to grams per cm^3^ is performed by using linear regression to relate the HU number of the vertebral bone to that of the compartments of the calibration standard.

In addition to measurements of bone density, QCT offers the possibility of measuring the height and the cross-sectional area of each vertebral body (20). The heights of the anterior, middle, and posterior portions of the vertebral bodies are measured on lateral scoutview, while the area of the vertebral body is calculated on CT scans corresponding to a ROI in each vertebral body, and excluding structures behind the most anterior margin of the spinal canal.

The recent application of QCT to assess the appendicular skeleton has significantly improved the ability to measure cortical bone. Three bone parameters can be measured by QCT in the appendicular skeleton, the cross-sectional area (cm^2^) of the bone, the cortical bone area (cm^2^) and the cortical bone density (21, 22). Appendicular bone measurements are usually performed at the mid-shaft of the femur. The scanning site is located by physical examination, and CT measurements are obtained from single 1.5 mm thick imaging scan at the mid-portion of the distance between the greater trochanter and the lateral condyle (21). The cortical bone density is measured using a calibration device, which is placed under the thigh. The procedure for calculation of bone density is similar to that described for the vertebrae. The outer and inner boundaries of the cortex are identified by specially developed software at the place of the maximum slope of the femoral profile through the bone. The area within the outer cortical shell represents the cross-sectional area, while the area between the outer and inner shells represents the cortical bone area. The mean CT numbers of the pixels within the inner and outer cortical shells provide the average density of bone (21).

As stated earlier, QCT measurements of bone are able to separately assess the density of cortical and trabecular bones. Moreover, the scan section has a definite thickness, which varies according to the region examined. Bone density measurements are expressed as grams per cubic centimeters, and are therefore true volumetric measurements. However, some limitations are present in CT bone measurements. The CT image is formed by thousands of pixels, small squares that have a different optical density according to the tissue they represent. The smaller the pixel size the better is the resolution of the image. Unfortunately, the size of the trabeculae in cancellous bone is frequently smaller than the pixel size, and therefore what is represented is not only bone, but also marrow. The optical density will then change according to the amount of prevalent tissue within the area of the pixel. This phenomenon is defined as volume averaging and is, therefore, a potential problem when measuring trabecular bone, or an extremely porous cortical bone. In children, QCT measurements at the midshaft of the femur reflect the material density of the bone (the amount of collagen and mineral in a given volume of bone), because the relative lack of porosity and the thickness of the cortex circumvent volume-averaging error (21). The minimum thickness necessary for an accurate density evaluation of cortical bone by QCT is 2-2.5 mm; below this threshold QCT values fall in a linear way relative to width. Above this thickness, the measured pixel represents the combination of the attenuation coefficients defined by the densities and concentrations of osteoid and mineral. While the nonmineral fraction may contribute to minor fluctuations in measurements of cortical bone density, QCT numbers are primarily based on the calcified bone fraction, which has a high attenuation coefficient (21). These measurements are analogous to in vitro determinations of the intrinsic mineral density of bone, which are commonly expressed as the ash weight per unit volume of bone (23). In children, the material density of cortical bone in the appendicular skeleton when measured by QCT remains fairly constant (21).

Calibration devices that are commercially available differ in the composition of materials used, and are designed for adult subjects. Special calibration reference phantoms have, therefore, been developed for children to provide precise measurements even in the very young (24).

The precision of QCT bone measurement is good in children. Coefficients of variation for determinations of cancellous bone density, vertebral body height, and vertebral cross-sectional area have been calculated as 1.5%, 1.3%, and 0.8%, respectively (25, 26). The coefficients of variation for repeated QCT measurements of cortical bone density, cortical bone area and the cross-sectional area of the femur range between 0.6 and 1.5% (27).

The current child with leptin deficiency offers a unique opportunity to assess the impact of leptin deficiency in children by measuring of bone mass by computed tomography of the spine and bone histomorphometry. Dr Gilsanz is an established investigator who has made substantial contributions to the determinations of bone in children using computed tomography. Although, anterior iliac crest bone biopsy is an invasive procedure, the morbidity is minimal. The bone histomorphometric analysis will allow to asses the role of leptin deficiency on the osteoblastic and osteoclastic activity as well as on indices of bone formation. Indeed, the fact that this child will receive replacement therapy with leptin provides a unique opportunity to determine the role of such agent on bone mass as well as indices of bone formation and resorption. In addition, such values will be correlated with serum and urinary markers of bone formation and resorption that will be measured at regular intervals during the first year of leptin therapy. At the end of the year, bone density and iliac crest bone biopsy after double tetracycline labeled will be repeated.

The committee may question the need for such an invasive procedure in a small child. It must be noted that leptin deficiency can be fatal. As described in the background section, most other children in this family died due to this genetic defect. The adult male patient has had osteopenia. Moreover, the effects of leptin replacement on bone in leptin-deficient children have have not been ascertained. As leptin is known to have an effect on bone, we believe it is medically important to document bone status before and after leptin treatment. A brief overview of the need to assess the effects of leptin on bone is provided here: It has long been known that there is a negative correlation between obesity and bone mass. Osteoporosis is one of the unusual situations where obesity is a protective factor (28). The relationship between adipose tissue and bone mass extends to the bone marrow, in which, there is a clear inverse relationship between adipogenesis and osteogenesis. Adipocytes and osteoblasts share a common stem cell precursor, and there appears to be preferential differentiation along the adipocyte lineage in different conditions such as aging and estrogen withdrawal. Bone histomorphometry in leptin-deficient humans has not been studied before. Given the fact that the male adult leptin-deficient patient has osteopenia, histomorphometric analysis of bone before and after leptin replacement therapy in this child would give us a unique opportunity to contribute to the current knowledge of the role of leptin on bone. Thus, the current study will determine bone mass and bone histomorphometry before and after one year of treatment with leptin replacement. This procedure would also contribute to the medical assessment of this child and would tell us whether leptin replacement alone is sufficient for his treatment or whether residual defects would require treatments other than leptin replacement.

Finally, because our preliminary results in adult patients and literature suggest that leptin may have a role in brain growth and development, we will conduct volumetric brain imaging studies in this patient at the UM Imaging Facility during the course of leptin replacement, ensuring safe exposure to radiation.

The dose of leptin for this study will be calculated to achieve 10% predicted serum leptin concentration based on age, gender and percentage of body fat (pharmacokinetic and pharmacodynamic data on file, Amgen Inc., Thousand Oaks, CA). Drug will be provided by Amgen Inc., Thousand Oaks, CA.

fMRI

We previously observed that leptin replacement to the three genetically leptin-deficient adults in this study altered the tissue composition, as measured by MRI (Matochik et al., 2005). We observed an increase in gray matter concentration after 6 months of treatment, and this effect was sustained for over a year. One area where this effect occurred was the anterior cingulate gyrus, which is activated by visual cues that trigger drug craving in individuals who have drug addictions (Breiter et al., 1997; Bonson et al., 2002; Brody et al, 2002; Childress et al., 1999; Garavan et al., 2000; Maas et al., 1998). The other areas that showed this effect were the cerebellum, and the inferior parietal lobule. All of the regions where we observed increases in gray matter concentration have been implicated in neural circuits regulating hunger and satiation in functional imaging studies (Tataranni et al., 1995). We hypothesize that leptin replacement reduces craving for food in leptin-deficient patients by an effect in the anterior cingulate gyrus.

The additional studies, comparing tissue composition and regional brain reactivity both on and off medication, will allow us to probe the mechanism by which leptin modulates regulation of food intake in leptin-deficient subjects who have reached to normal body weight by leptin replacement therapy.

This work will build on knowledge of the circuitry important to craving states and on our previous observation that one primary component of this circuitry is affected by leptin replacement therapy. We anticipate that our *f*MRI study will reveal abnormalities (hyper-responsivity) in activation when the patients are not receiving leptin replacement therapy, and that activation will be reduced with a concomitant reduction in hunger or (cure-induced) food craving when treatment is administered.

**Measuring Brain Function with *f*MRI.** We will pair *f*MRI with cognitive/behavioral tasks to test brain function. The approach depends on the fact that changes in the magnetic resonance signal accompany parallel changes in brain activity. We will use the BOLD (Blood Oxygenation Level Dependent) approach, (Kwong et al, 1992, Ogawa et al, 1992) which reflects local increases in blood flow that accompany increases in local brain function,(Roy et al, 1890) and takes advantage of the different magnetic susceptibilities (χ) of the oxygenated and deoxygenated forms of hemoglobin. The greater χ of deoxyhemoglobin produces a local perturbation of the magnetic field and, therefore, a decrease in the amplitude of the MR signal.(Cohen, 1997) Because the extraction fraction of oxygen across the capillaries decreases in the face of increased blood flow, the oxygen concentration increases in the post-capillary venules.^10^ The post-capillary oxyhemoglobin increases and so, therefore, does the MR signal. (Kwong,1992).

**12.2** **Methods and Procedures:** Using **non-technical, lay** language, please answer the following:

12.2.1 Please describe in detail the study design and all study procedures, in order of sequence and timing, including frequency of visits, duration of visits, length of subject participation, specific measures to be used, etc.

This is a carefully designed, prospective clinical study of the effect leptin-replacement treatment in children. The subject will undergo a comprehensive intake assessment, and then follow-up assessments and treatment performed under the protocol. Leptin replacement treatment will be given for 26 weeks. During this time subject will also undergo Chest CT and hand-wrist X-rays along with bone biopsy and blood and urine samples to test immune system functions.

Because of the small size of the child, and young age, we will perform tests that are minimally invasive but which can provide scientifically important data regarding the phenotype of leptin deficiency.

The child would be studied at the UM General Clinical Research Center. This study will involve blood collection at baseline and longitudinally during the course of leptin replacement treatment. The total amount of blood drawn will not exceed 3 ml per kg in a 6-week period. Specifically, tests and procedures will include:

Immunology

Screening tests (CBC with ESR and CRP, chem. panel, IgG, M, A, E, and IgG subclasses, CH 50, C3 and C4 levels, mannose binding protein, HLA typing, Howell Jolly bodies for splenic deficiency, proliferative responses to PHA, Tetanus and Candida), urinalysis, sinus, lateral pharynx, and chest x-rays (if abnormal CT scans)

Other tests

Tests for antibody (tetanus, diphtheria and H. Influenzae type B antibodies, baseline 12 serotype Pneumococcal antibody panel), complement, and phagocytic function, Rhodamine flow test for oxidative function

Tests for T-cell immunity (T, B and NK cell subsets CD3, CD4, CD8, CD16/56, CD19).

Flow cytometric analysis to identify mononuclear cell subsets including naive T cells subsets, and the TREC PCR assay to quantify thymus activity

Screening tests will be performed two weeks before treatment. Other tests will be performed at baseline and throughout the treatment (baseline, weeks2, 6, 24, and 52)

Thymic size by CT-scan: to determine if leptin deficiency has lead to a reduction of thymic mass, and to monitor the effects of leptin treatment, we plan to quantify the amount of thymic tissue that remains in the anterior chest. Computed tomography has been used in a number of studies to monitor changes in thymic mass in HIV infected children and adults, and can be completed in minutes, without use of sedation, using a spiral CT scanner. Magnetic resonance imaging can provide similar information, but requires a more lengthy period of scanning. We plan to use CT scanning, employing either a GE or Siemmens scanner, both capable of 1 second scans. The assumed protocol is: scan thoracic inlet to mid chest., 3 mm collimation, pitch 1, 120 kVp, 250 mAs. Following the scanning, a tridimensional (3D) reconstruction with volume calculation will be performed at the imaging laboratory of UM Department of Radiological Services. After 6 months of leptin treatment, CT quantitation of thymic volume will be repeated.

Immunizations - if any of the antibodies for tetanus, diphteria and H. Influenza, we suggest giving him a single booster vaccines (as routinely recommended for all non-protected children and rechecking titers one month later. This is standard pediatric practice. There are very rare side effects with these vaccines such as fever and local swelling, but in general they are well tolerated.

We also suggest giving the patient pneumovax at baseline and checking Pneumococcal antibody titers in 1 month. The pneumocococcal polysaccharide vaccine is a killed vaccine containing 23 serotypes of pneumococci used to immunize special groups of children over age 2 yrs who are likely to have problems with pneumococcal ear, sinus, blood, and lung infections such as partially immunodeficient children, sickle cell patients, patients receiving immunosuppressive drugs, or normal children with too many infections. It is a safe vaccine approved for children although local and febrile reactions may occur. It is an excellent antigen to test for subtle immune defects since it is not a strong antigen and identifies children who have defective antibody responses to polysaccharides.If they make antibodies to these serotypes they are not antibody immunodeficient. The response is measured before and 1 month after vaccine.

Endocrine function

Blood draws for hormones (withdrawing small amounts of blood at 08:00 h, 13:00 h, 19:00 h, and 21:00 h) to examine day/night variations in hormone levels including ACTH, cortisol, TSH, T3, T4, GH, glucose, insulin and C-peptide and related substances). Blood will be drawn four times in a day, two times in a week during the acclimatization period and throughout the treatment (weeks -2, -1, 0 and weeks 4, 28, 52) as shown in the flowchart.

Diet

The child would be allowed to ad libid so that the effects of leptin on food intake and nutrient choice could be documented. Food records will be obtained at during acclimation and through the treatment. Food diaries will be recorded at the General Clinical Research Center by the child’s mother and dietary personnel, and will be reviewed by research dietitian. All completed records will be analyzed using the nutrition analysis software Nutritionist Pro version 1.2.2 (First DataBank, Inc.).

Activity

Twenty-four-hour physical activity level of the child will be assessed at baseline and through the treatment period by an accelerometer-based activity monitor (Actiwatch®, Mini-mitter Company Inc., Bend, OR, USA)

Bone/body composition evaluation

Bone and body composition studies will include quantitative CT, bone resorption and formation markers, and bone biopsy.

Bone and body composition will be assessed by CT at baseline and at 6 and 12 months of leptin replacement treatment.

Technique of CT. The child will be assessed by CT using the scanner (General Electric Hilite Advantage, Milwaukee, WI) and the mineral reference phantom for simultaneous calibration (CT-T bone densitometry package; General Electric). In the axial skeleton identification of the sites to be scanned will be performed with lateral scout views and two measurements will be obtained at the 1st, 2nd and 3rd lumbar vertebrae: the cross-sectional area at the midportion of the vertebral body (mm2) and the density of cancellous bone at this level, which is defined as the amount of bone and marrow in milligrams per cubic centimeter per pixel (the CT unit of measurement). In the femur, location of the site to be scanned will be done by physical examination and the following structural parameters will be measured: the cross-sectional area (mm2) the cortical bone area (mm2) and the cortical bone density (mg/cm3). The coefficients of variation for repeated CT measurements of cancellous bone density, cortical bone density, cortical bone area and cross-sectional areas of the femur and vertebral body range between 0.6 and 1.5% (29, 30).

From the same CT cross-sectional images obtained at L3 and the midshaft of the femur, the areas of paraspinous and thigh musculature (mm2) will be determined. At these sites, the cross-sectional areas of fat (mm2) will also be obtained. The coefficients of variation for repeated CT measurements of fat and muscle in the thighs and abdomen are between 1-2% (31-33).

The time required to complete CT scans in an individual patient is approximately 10 minutes. CT measurements will be obtained at 1.5 or 10 mm thickness, 80 kVp, 70 milliamperes, and 2 seconds. Radiation is 100-150 mrems (10-15 mJoules/kg) localized to the 10 millimeter-thick section of imaging in the midportions of the L1, L2 and L3 vertebral bodies, and the 1.5 mm-thick section at the mid-thigh (34). The effective radiation dose is approximately 10 mrem (0.10 mJ per kilogram) and this dose includes that associated with the scout view (34). The scanner is calibrated with a hydroxyapatite phantom of known hydroxyapatite density (164 mg/cm3) daily.

Several comprehensive reviews of the risks associated with CT determinations in children and young adults have been done by us and other investigators (34, 35). They conclude that the radiation exposure from CT measurements is related to the technique employed and can be as low as 100 mrem (1.0 mSv) localized to the region of interest without exposure to the gonads, lens or thyroid. The total body equivalent dose of radiation is approximately 10 mrems (100 µSv), and this figure includes the radiation associated with screening digital radiographs used to localize the site of measurement (34, 35). This amount of radiation is far lower than that associated with other CT imaging procedures, accounting for the wide range of published figures for the radiation dose associated with CT measurements. By comparison, a round-trip transcontinental flight in North America exposes a child to roughly 8 mrems (80 µSv) of ionizing radiation (34, 35). Therefore, bone measurement determinations using CT do not expose children to amounts of ionizing radiation that deviate from the amount that constitutes part of their normal life experience. The most recent report of the National Commission on Radiation Protection provides an estimate of the natural background whole body radiation exposure which is approximately 350 mrem per year, or one mrem per day (35, 36).

*Summary of whole body equivalent doses in millirems (mrem)*

| **Exam** | | **Age (years)** | | |
| --- | --- | --- | --- | --- |
|  |  | **5** | **10** | **20** |
| Chest X-ray |  | 4.4 | 3.9 | 3.2 |
| Lumbar Spine | Anteroposterior | 2.1 | 2.0 | 1.8 |
|  | Lateral | 13.1 | 12.1 | 11.2 |
| DXA | PA Spine | 0.1 | 0.1 | 0.1 |
|  | Lat Spine | 0.3 | 0.3 | 0.3 |
|  | Proximal Femur | 0.1 | 0.1 | 0.1 |
|  | Whole Body | 0.4 | 0.4 | 0.4 |
| CT | Scout View | 4.8 | 4.5 | 4.1 |
|  | Axial Scan | 0.9 | 0.9 | 0.8 |
| Natural Background (per month) | | 22 | 18 | 11 |
| Transcontinental Flight | | 4 | 3 | 2 |

*Adapted from references (34, 35, 37)*

New research on how cells fix DNA damage has led scholars to adopt a less stringent estimate of radiation risk. Based on the scarcity of human data on low-level effects, for the last 25 years, regulators relied on a “linear no-threshold model” to restrict radon levels in drinking water, exposure for nuclear power workers, and limits on x-rays, among other health standards. The linear no-threshold model “was adapted specifically on a basis of mathematical simplicity, not from radio-biological data, during the period between 1950 and 1964 as the only practicable mathematical approach to estimates of the maximum effects of world-wide fallout from atmospheric weapons testing” (36). Available data now indicates that there is a threshold for radiation damage and cellular studies even go so far as to suggest that low doses may help prevent cancer by stimulating DNA repair (36). Thus, the long-running belief implied that no amount of radiation is too small to cause harm and that the DNA damage increases directly with a persons exposure is unproven (36, 38). It should be noted that, based on our current knowledge of the risk of low levels of ionizing radiation from radiographic diagnostic equipment, the American Association of Women Radiologists, the American College of Radiology, and the British Institute of Radiology have established their recommendations pertaining to acceptable levels of radiation exposure for medical personnel. Accordingly, women radiologists do not interrupt their professional activities during pregnancy although the levels of radiation exposure to both the mother and the fetus are considerably higher than those from natural background radiation (39).

CT measurements of bone, muscle and fat. CT studies will be performed in the abdomen with the same scanner (model CT-T 9800,General Electric Co., Milwaukee, WI), the same reference phantom for simultaneous calibration, and specially designed software for fat, muscle and bone measurements. Identification of the sites to be scanned will be performed with lateral scout views, followed by cross-sectional images obtained from the midportion of the first through the third lumbar vertebrae at 80 kVp, 70 milliamperes, and 2 S. The time required for the procedures is approximately 10 minutes and the radiation exposure of this limited CT exam is 100-150 mrem (1-1.5 mSv) localized to the midportions of the first three lumbar vertebrae; the effective radiation dose is approximately 4 mrem (34).

For the purposes of this study, subcutaneous fat volume will be defined as the average amount of adipose tissues located between the skin and the rectus abdominus muscles, the external oblique muscles, the latissimus dorsi muscles and the erector spinae muscles at the midportions of the 1st, 2nd and 3rd lumbar vertebrae. Visceral fat volume is defined as the intraabdominal adipose tissue surrounded by the rectus abdominus muscles, the external oblique muscles, the quadratus lumborum, the psoas muscles and the lumbar spine at the midportions of the first three lumbar vertebrae and consists mainly of perirenal, pararenal, retroperitoneal and mesenteric fat. Total abdominal fat volume is defined as all subcutaneous and visceral adipose tissues at the midportions of the first three lumbar vertebrae. Paraspinous musculature volume is defined as the sums of the volumes of the erector spinae muscles, psoas major muscles and quadratus lumborum muscles from at the midportions of the first three lumbar vertebrae. Vertebral cross-sectional area is defined as the mean cross-sectional dimensions of the first three lumbar vertebral bodies. Vertebral height is defined as the mean height of the first three lumbar vertebral bodies. Vertebral cancellous bone density is defined as the amount of bone and marrow in milligrams per cubic centimeter per pixel, the CT unit of measurement at the midportion of the first three lumbar vertebrae. The coefficients of variation for repeated measurements for each of these traits were determined using images from three children and ranged from 1.5% to 3.5%.

Bone biopsy. Iliac crest bone biopsy is a well-established and frequently used technique at UM. Most biopsies are done as an outpatient procedure and this approach has been quite successful, and no patient has refused to undergo a second bone biopsy because of an adverse experience with the bone biopsy procedure. Bone biopsy of the child in this protocol will be performed baseline and after one year of leptin replacement treatment. Specimen of bone will be obtained from the anterior iliac crest after double tetracycline labeling, and quantitative histomorphometry of bone will be completed as previously described from the e UM GCRC Core laboratory.

Bone markers. Bone resorption and formation markers will be assessed at baseline and 3,6,9 and 12 months of leptin replacement treatment. These will include serum Ca, P, B-ALP, osteocalcin, urinary Ca, Cre, P, hydroxyproline, deoxypyridinoline. We will also measure PTH, 25 hydroxy- and 1,25 dihydroxy-vitamin D levels.

Brain Imaging

Brain imaging by magnetic resonance imaging (MRI) will be performed at baseline, 6 and 12 months. The MRI scan is a minimal risk and there are no known risks associated with the procedure.

ƒMRI Procedures

The sets of MRI tests (one structural, one functional) will be scheduled as follows: 1) 1 set before leptin therapy begins, 2) 1 set two weeks after leptin therapy begins.The first set of tests will act as a control for the later tests and there are 2 structural MRI’s and 2 functional MRI scans for a total of 4 scans. Each set of MRI tests will be done on sequential days . Each functional MRI, and Structural MRI session will last less than 45 minutes.

We will accompany subjects to the brain mapping building for scanning. After each session, subjects will return to the GCRC in-patient facilities, where they will be housed until they complete their participation.

Participants will lie on the bed of the MRI scanner and will be given headphones and earplugs. They will also be given an MRI-compatible video system (goggles) through which they can watch movies of their choice (during preliminary scans and positioning). In some cases, a cloth tape or pads on the sides of the head may be used to limit head motion. During the fMRI procedures (typically 30 – 45 min), the participants will be asked to relax and to rate how hungry they feel in response to pictures we will show them (either pictures of food or neutral pictures, such as landscapes).

The MRI equipment makes a variety of noises during scanning (e.g., repetitive tapping, beeping noises). These are all below the threshold for hearing damage, but we will use ear protectors and headphones that offer an additional 45-60 dB of attenuation, so that the noise that the subject experiences is not excessively loud. Most of the scans will require acquisition of multiple images, each lasting from 1 to 15 min. The subject will be asked to remain still during the imaging period, but can move more freely during the 2-5 min that separates the imaging series.

After completing the imaging, the subject will be removed from the scanner, and we will ask about any adverse or unexpected reactions that may have taken place during scanning (none have been reported so far), and administer questionnaires related to mood states.

Neuropsychological assessment

The child will undergo neuropsychological testing at baseline and three times during the 12-month leptin replacement treatment (see flowchart). This will be done by experienced clinical neuropsychologists with appropriate language interactions in Turkish. These are minimally invasive procedures and are not expected to be associated with any adverse reactions.

In the process of the research the subject will be videotaped and/or photographed, and/or audio-taped. The tapes or photographs will be used for teaching and/or research purposes to enable detailed analysis of the testing session and may be used in research presentations. The subject will have the right to review the tapes/photographs made as a part of the study to determine whether they should be edited or erased in whole or in part.

Procedure flowchart

| **Weeks** |  | **1-12** | | | **13-28** | **29-36** | **37-51** | **52-** |
| --- | --- | --- | --- | --- | --- | --- | --- | --- |
| **Visit** | **Screening exam**  **TURKEY** | **1-4**  **UM**  **GCRC** | **5-8**  **UM**  **GCRC** | **9-12**  **outpatient follow-up** | **outpatient follow-up** | **UM GCRC** | **outpatient follow-up** | **UM**  **GCRC** |
| Physical exam | ✓ | ✓ | ✓ | ✓ | ✓ | ✓ | ✓ | ✓ |
| Urine and blood collection  for routine tests | ✓ |  |  |  | ✓ |  | ✓ |  |
| **Endocrine** |  | | | | | | | |
| ACTH, cortisol, TSH, T3, T4, GH, glucose, insulin, C-peptide, and related substances  4 times over 24 h |  | ✓ |  | ✓ |  | ✓ |  | ✓ |
| **Immunology** |  | | | | | | | |
| Screening tests |  | ✓ |  |  |  |  |  |  |
| Tests for antibody, complement and phagocytic function |  | ✓ | ✓ | ✓ |  |  |  |  |
| Tests for T-cell immunity |  | ✓ | ✓ | ✓ |  |  |  |  |
| Flow cytometry/TREC PCR |  | ✓ | ✓ | ✓ |  |  |  |  |
| Thymus CT |  | ✓ |  |  |  | ✓ |  |  |
| Ab titers check |  | ✓ | ✓ |  |  |  |  |  |
| Immunization |  | ✓ |  |  |  |  |  |  |
| **Bone /body composition** |  | | | | | | | |
| Hand-wrist X-ray |  | ✓ |  |  |  | ✓ |  | ✓ |
| Markers |  | ✓ |  | ✓ |  | ✓ |  | ✓ |
| QCT |  | ✓ |  |  |  | ✓ |  | ✓ |
| Bone Biopsy |  | ✓ |  |  |  |  |  | ✓ |
| **Brain imaging** |  | | | | | | | |
| **Magnetic resonance imaging** |  | ✓ |  |  |  | ✓ |  | ✓ |
| **Neuropsychological evaluation** |  | | | | | | | |
| Neuropsych testing |  | ✓ | ✓ |  | ✓ | ✓ |  | ✓ |
| Start of daily Leptin  injections |  |  | ✓ |  |  |  |  |  |

# Media Interview

# It has never been the policy at UM to allow a representative of the media to contact a patient directly. This study focuses on a single pediatric research subject. Therefore, if the subject’s parent consents, we shall provide the parent with the contact information for a UM-hired videographer and allow the subject’s mother to initiate contact if she so desires. In light of language issues, however, the parent also will be given the option of having the Health Sciences Media Relations officer arrange the interview on her behalf, if she prefers.

When a representative of the media requests to interview the subject, we will:

- Inform the subject’s parent by phone or at a regularly scheduled visit of the videographer’s interest in conducting interviews;
- Provide the subject’s parent with information, discuss the video request, explain the interview process, and answer any questions that the subject and his parent may have.
- Provide the subject’s parent with a copy of the video interview consent form to review, allowing them sufficient time to review the form;
- Assure the subject and his parent that their decision to participate in the media interview will in no way affect the subject’s treatment;
- Once I have obtained written consent from the subject’s parent, the Media Relations Office will provide her with the appropriate contact information for the videographer. The office will also provide her with the option of arranging the interview on her behalf, if she prefers this option.

1. Following is a script that will be used to inform subjects about media interest in

the research:

- A freelance videographer has expressed an interest in a study in which you are participating at UM. She would like to interview your son and you in this study in order to inform the public about the research.
- I would like to assure you that your decision whether to participate or not in this interview will have absolutely no impact on your participation in this study, and will in no way affect your relationship with the study staff or UM.
- If your son and you decide you would like to be interviewed, and the interview will be at UM, we will do everything we can to assure that the interview takes place on one of your regularly scheduled clinic visit days and near the time of your clinic appointment so that it is as convenient for you as possible.
- If your son and you decide you would like to participate in the interview, you will be given the name and telephone number of the videographer, and you will make the contact yourself. No media representative will have access to your telephone number unless you choose to give your number to the interviewer.
- Take some time to think about this. If you decide you would like to participate in an interview, or if you have questions that you would like to have answered before you make your decision, please call Dr. Julio Licinio.

Leptin-replacement treatment

Study Medication (A-100)

Other Names: Recombinant-methionyl Human Leptin (r-metHuLeptin)

Formulation:

A-100 will be supplied as a single-use only glass vial containing a lyophilized cake, prepared in a formulation buffer consisting of 10 mM glutamic acid, 2% glycine, 1% sucrose, and 0.01% Tween 20 to pH 4.25. Upon reconstitution with 2.2 ml of sterile water, the cake will yield a 5 mg/ml concentration of A-100.

Storage Conditions and Stability:

The study drug must be stored in a secure location under controlled conditions at the study site or pharmacy before dispensing to subjects. A-100 must be stored at 2-8o C prior to reconstitution. It is recommended that the refrigerator be connected to a back-up power source, and a temperature alert system. Amgen must be notified if any test material is exposed to excessive or uncontrolled temperatures; possible replacement of the affected material will be considered. Study staff must be instructed in proper storage of the study drug.

Storage Temperature Monitoring:

Records of the storage conditions should be maintained throughout the study by either a continuous temperature recording system, a regularly maintained temperature alarm system or by regular visual inspection of a calibrated thermometer placed inside the refrigeration unit.

Expiration Dating:

As per standard practice for experimental biologic pharmaceuticals, Amgen will conduct periodic stability assays to monitor product stability and determine appropriate expiration dating of the study drug. The appropriate Amgen representative shall communicate this information to the investigator.

Drug Dosage and Administration:

The study seeks to examine the effects of leptin replacement in lone eptin deficient child. R-metHuLeptin will be injected subcutaneously once a day at approximately the same time in the evening at dose of 0.028 mg/kg/day The starting dose of the medication for the child is based on Farooqi’s study of 2 Pakistani’s cousins (8).

Data Collection, Storage and Confidentiality:

All blood analyses and other test results will be kept in locked cabinet files and restricted-access computers. They will be kept separate from records with subject's name and other personal information. The only people who will know that the patient is a research subject are members of the research team and, if appropriate, their physicians and nurses. No information about the patient will be disclosed to others without his mother's written permission, except if it is necessary to protect his rights or welfare (for example, if he is injured and need emergency care), or if it is required by law. When the results of the research are published or discussed in conferences, no information will be included that would reveal the patient's identity. If photographs, videos, or audiotape recordings of them will be used for educational purposes, his identity will be protected or disguised. All data will be kept in locked files, and patient will be identified by codes when the data gathered in this procedure is presented or published.

Authorized representatives of the Food and Drug Administration (FDA) or a funding agency, such as the National Institutes of Health may need to review records of the subject. As a result, they may see his name; but they are bound by rules of confidentiality not to reveal his identity to others.

Payment for Participation:

Amgen will provide leptin free of charge to subjects during their participation in this study. National Institute of Health will cover the research costs of this study. There is no special participation fee in this study.

Subjects will receive monetary compensation for their participation in the fMRI part of the study. They will be paid $40 for each MRI session, and these sessions will each last less than one hour. If a session takes more than 45 min, there will be a break. We estimate that if a subject takes part in all of the MRI scans, he or she could earn a maximum of $160 for this part of the protocol.

Financial Obligations of the Subjects:

Neither the subjects nor their insurance company will be billed for their participation in this research. Other medical care, which is not scheduled for this study, may be charged to the patient or his insurance company.

Emergency Care and Compensation for Research-Related Injury:

If a subject is injured as a direct result of the research procedures not done primarily for one’s own benefit, he or she will receive treatment at no cost.

12.2.2 If applicable, please identify and distinguish between those procedures that are standard treatment versus those that are experimental/research specific:

There is no other treatment for individuals with a Mendelian recessive leptin gene mutation consisting of a C-T substitution in codon 105 of the leptin gene, resulting in an Arg-Trp replacement in the mature protein.

12.2.3 If applicable, please describe any therapeutic alternatives that may exist:

There is no other treatment for individuals with a Mendelian recessive leptin gene mutation consisting of a C-T substitution in codon 105 of the leptin gene, resulting in an Arg-Trp replacement in the mature protein. Therapeutic alternatives to this study are the other available treatments for childhood obesity such as diet and exercise.

**12.3** **Deception: Complete this question only if using deception as a study design method.**

12.3.1 Please describe in detail the nature of the deception and explain why this is necessary for the research.

N/A

12.3.2 Please state how, when and by whom the research participants will be debriefed.

N/A

**12.4** **Risk/Benefit Assessment:**

12.4.1 Describe the nature, degree, and if available, expected frequency of all potential economic/financial, legal, physical, psychological, social or other risks to which research participants may be exposed as a result of their participation in this research.

The known risks, inconvienences or side effects involved in this study are the following:

a) Side effects of Leptin:

The most frequently reported side events in studies of A-100 have been skin reactions (injection site reactions) at the site where the drug is injected. These reactions include bruising, redness, pain, itching, inflammation, swelling, dark spots on skin, and lumps under the skin. Other frequently reported adverse events have been headache, fatigue, nausea and influenza-like symptoms. There may be additional risks such as potential allergic reaction to the study drug. There have been less frequent reports of generalized rashes, hives, and in rare instants, swelling of the lips and eyes..

b) Blood loss: The patient will lose no more than 150 ml or 3.5 ml per kg in an 6 week period

c) Blood drawing: The patient may experience pain or discomfort during the insertion of needles. The risks of infection from this procedure will be minimized by the use of careful sterile techniques

d) Exposure to radiation:

We are exposed to radiation every day of our lives from both natural (sun, earth, etc.) and man-made sources. The average radiation dose from these sources for residents of the United States is about 100 mrem per year. The radiation exposure from the limited CT scan is considered minimal and is less than that of one chest x-ray (or less than the radiation exposure during a round-trip flight across the United States). The total body equivalent dose of radiation is approximately 10 mrems (100 µSv). This amount of radiation is far lower than that associated with other CT imaging procedures.

e) Bone biopsy:

Risks associated with bone biopsy procedure are local pain, possible bleeding and infection. These symptoms are usually so mild that people undergoing bone biopsies usually resume normal walking and other activities of daily life the same day of the biopsy. There may be a slight tenderness at the site of the biopsy the day after; this is usually relieved with plain Tylenol. In our experience these symptoms rarely develop.

f) Blood drawing

The remote risk of infection, inflammation, blood clotting, or continual bleeding at the site of needle insertion are the risks of blood drawing. Your child may experience pain, bruising, lightheadedness and fainting. In our experience these symptoms rarely develop.

g) MRI Scan

Although there are no known risks associated with the MRI scanning procedure. MRI scanner technology is relatively new, there could be unknown long-term risks. However, in the studies performed so far with similar exposures, there have been no other significant risks reported in either animals or humans.

*MRI Scanning:* There are no known adverse affects resulting from exposure to the MRI scanning procedure *per se*; however, the magnetism of the machine attracts certain metals. Therefore, if a subject were scanned without making the investigator aware of the presence of a device containing such a metal (e.g., cardiac pacemaker, artificial heart valve, implanted infusion pump, cochlear implant, or spinal cord stimulator, aneurysm clips, metal prostheses, joints, rods, or plates) in his or her body, the magnetism could affect or stop the device from working properly. Subjects will be queried as to whether they have any of these devices implanted, and if so, will be excluded from participation. Subjects will also be informed of the consequences that could result in undergoing an MRI scan with such an implanted device present. The "metal" in dental fillings is less susceptible to magnetism and is therefore allowed.

The MRI scanning procedure requires that the subject be confined in a small, partially enclosed space. During the MRI, subjects may experience some anxiety or claustrophobia associated with this confinement. All efforts will be made to assist the subject in remaining calm and as comfortable as possible.

The sound of the MRI scanner can be loud, but will be reduced by special earplugs. Viewing pictures of food may cause hunger or food craving.

There is a very small possibility that a brain abnormality (i.e. cyst, tumor, etc.) may be discovered from the scan images. In this case, the image will initially examined by a radiologist. The radiologist will alert the PI and study physician and give them his radiological interpretation. The study physician will contact the research participant to discuss the scan for potential referrals to local medical facilities (at subject’s cost). A possible associated risk may be anxiety from the notification of the abnormality. However, since we are conducting a research study and not diagnosing abnormalities in subjects’ scans, there are limitations on interpretations. It is possible that we may not detect some abnormalities under our examination. Participants will be informed of this limitation and advised to seek their primary physicians if they have any health concerns outside the scope of the study.

h) Neuropsychological Testing

Your child may experience emotional distress in the course of detailed, structured, psychiatric interviewing, as well as boredom and fatigue. This will be minimized by our performing the interviews in a sensitive way by experienced members of our research group who have had specific training on structured psychiatric interviewing.

**Risk Classification: T**he present study risk cannot be classified as minimal.

12.4.2 Describe in detail any measures in place to minimize or protect against the exposure of research participants to these risks. Discuss all provisions for intervention in the event of adverse events of adverse effects.

The risks from blood drawing procedure will be minimized by the use of careful sterile techniques performed by experience research staff. Primary Investigator or another participating investigator will be present during the whole procedure in the event of adverse effects to the subject.

All potential risks and discomforts will be minimized by continuous monitoring by participating investigators.

The FDA considers MRI devices to be of non-significant risk if they meet the following criteria (we summarize only the relevant portions of this document):

## Field strength less than 4 Tesla

2) RF power deposition (measured as the specific absorption rate or SAR) to the patient is limited by law, and by the instrument, to less than 3 W/kg in the head. Notably, human imaging with SAR of double that has been demonstrated to be safe in normal subjects[1].

3) Any time rate of change of gradient fields (dB/dt) sufficient to produce severe discomfort or painful nerve stimulation.

4) A-weighted root mean square (rms) sound pressure level greater than 99 dBA with hearing protection in place

Under no circumstances will our instrument be operated outside of these non-significant ranges on human subjects.

With respect to MRI, the screening process described previously will reduce the small risk of metal objects being dislodged. Headphones and ear protectors will be used to reduce any discomfort that might be associated with the scanner noise. The use of headphones and video systems will minimize the potential discomfort of claustrophobia.

12.4.3 Describe the potential benefits to the subjects as a result of participating in this research.

We expect that the weight and body composition of the patient may be changed to that of a normal boy during the course of the leptin treatment. Additionally, endocrine and immune dysfunction may be normalized.

12.4.4 Describe the potential benefits to society as a result of this research.

The results we obtain may give us information on how leptin affects other hormones and systems in children. This could lead to more effective treatment in other types of leptin deficient patients, for example leptin deficiency due to a leptin receptor mutation.

12.4.5 **Risk/Benefit Ratio:** Please discuss why the risks are reasonable in relation to the potential benefits.

There is no unreasonable risk in this study. The side effects of leptin are minimal and the boy's weight, body composition and endocrine levels could be returned to that of a normal child, which could greatly improve their quality of life.

**12.5** **Data Safety Monitoring Board:** Complete these questions only if an internal and/or external DSMB has been established to provide additional oversight or monitoring of this research .

12.5.1 Describe your external DSMB.

N/A

12.5.2 Describe your internal DSMB. Please include details on its operation, membership, and reporting procedures.

# Data and Safety Monitoring Plan

This is a GCRC study. All GCRC studies are required to have a Data and Safety Monitoring Plan. A strength of this application is that the P.I., Dr. Julio Licinio, is the Associate Program Director of the UCLAGCRC, and he is also a member of the UCLA GCRC Data and Safety Monitoring Board. This experience will greatly enhance the Data and Safety Monitoring aspects of the proposed research studies.

## a. Reporting

**a.i. Adverse Events Definition:**

An adverse event is any new, undesirable medical occurrence or change of an existing condition in a subject that occurs during or after treatment, whether or not considered to be product-related. If the adverse event is sufficiently severe in the investigator’s judgment, the subject should be removed from treatment and a termination assessment performed. The subject should be given appropriate care under medical supervision until symptoms cease.

**a.ii. Serious Adverse Events:**

A serious adverse event is defined by the FDA as any experience that suggests a significant hazard and includes any event that:

- Is fatal;
- Is immediately life-threatening;
- Requires or prolongs hospitalization;
- Is a persistent or significant disability/incapacity;
- Is a congenital anomaly or birth defect;
- Is an other significant medical hazard.

### a.iii. Deaths

All deaths on study must be reported to the UM Internal Review Board (IRB) within 1 working day of notification of the death. Because Leptin is administered under an FDA IND issued to me, the FDA will also be notified within 1 day. In addition, Amgen, which provides recombinant human leptin for the study, will also be notified of all deaths within 30 days of last investigational drug dose and/or deaths occurring out to the last formal follow-up observational period, whichever is longer. For all deaths, autopsy reports (if applicable) and relevant medical reports will be sent to Amgen Inc.

**a.iv. Serious Adverse Event (SAE) Reporting Procedures:**

Adverse events that are deemed serious by the investigator (irrespective of suspected causation) should be reported according to UM IRB guidelines.

Unexpected and related fatal or life-threatening events are reported to FDA by phone or FAX within 7 calendar days. Written report should be sent to FDA within 15 calendar days for any serious unexpected adverse events for which there is a reasonable possibility that the event may have been caused by study drug(s). Any follow-up information that is significant to the report must also be sent to FDA within 15 calendar days of receipt of the new information.

All adverse events occurring during the study, whether or not attributed to study drug, should be included in the investigator’s annual IND report to FDA. A copy of any 7 or 15-day reports sent to FDA related to Leptin will also be sent to Amgen’s International Clinical Safety Department (by Fax or mail) within 24 hours of notification or discovery of the event.

Julio Licinio

Chairman of the Department of Psychiatry

Miller School of Medicine

University of Miami

UM/JMH Mental Health Hospital Center

1695 NW 9th Avenue, Suite 3100

Miami, Florida 33136

Phone: (305) 355-9105

FAX: (305) 355-9099

Email: jlicinio@med.miami.edu

**b. Study Drug Management:**

**b.i. Initial Drug Shipment and Re-Supply:**

A signed and completed Drug Request Form must be faxed, at least 3 days prior to the expected delivery date, to Clinical Logistics, Amgen, Inc., 805-499-9697. Drug to be returned at the end of the study should be counted and placed in a box clearly marked with EXTRAMURAL on the outside of the box and sealed with tamper evident tape. Accompanying paperwork should be attached to the outside of the box. The drug is shipped to Amgen Inc., Returned Clinical Drug, Room 124A, One Amgen Center Drive, Thousand Oaks, CA 91320.

**b.ii. Supply of Drug:**

The study test drug will be shipped to the investigator's institution and should be checked for amount and condition of the drug received. This data will be entered into the proof of receipt letter. The proof of receipt letter should be faxed to Amgen Clinical Logistics at the number indicated on the letter and the original retained in the pharmacy files.

**b.iii. Drug Accountability:**

**• Directions for Handling the Product Receipt Forms:**

• The following forms should accompany each shipment of study drug to a study center:

* *Inventory Form:* An invoice listing the quantity, and name of the study drug. If there are discrepancies please record this information on the Product Receipt Checklist Fax Form and notify Rich Coatney as the supply contact person at Amgen.

* *Product Receipt Checklist Fax Form:* The pharmacist or designee receiving the shipment should review and answers all questions indicated on the forms. Forms should be completed and faxed to Amgen Clinical Logistics; to the fax number specified on the form, on the day the shipment is received.

* *Proof of Receipt Letters:*

Sign and date the Proof of Receipt Letter, retain a copy for the study records, and return the original to Amgen Inc. per the included instructions.

* *Schedule of treatment:*

Patients will be treated with leptin for 2 months.

**Data Management:**

Physiological, demographic, and clinical data will be collected on each subject. The Data Coordinating Center at UM will be responsible for receiving, editing, quality monitoring, storing, and analyzing all data. The Data Coordinating Center at UM will keep records of data generated in their site. The P.I. and the UM Data Coordinating Center will have access and maintain the integrity of the entire data collection.

**c.i. Supervision of Data Analysis:**

A strength of this application is the fact that data analysis will be supervised both by the PI and by the study coordinator Dr. Joao Vicente Busnello.

**12.6** **Inclusion/Exclusion Criteria:**

12.6.1 What are the criteria for inclusion of participants in the research?

Children with a functional leptin gene mutation from a consanguineous Turkish family. Only one leptin-naïve child from this family is alive and eligible.

12.6.2 What are the criteria for exclusion of participants from the research?

N/A

**12.7** **Recruitment Procedures:**

12.7.1 Describe in detail all recruitment strategies for each participant group selected for this research. Explain who will approach the participants; how and when will the participants be approached?

In 1998 Dr. Metin Ozata’s group at the Gulhane Military Medical School in Ankara, Turkey, in collaboration with Dr. Strosberg’s group at the University of Paris (VII) - Institut Cochin de Genetique Moleculaire identified two adult Turkish individuals with a Mendelian recessive leptin gene mutation consisting of a C-T substitution in codon 105 of the leptin gene, resulting in an Arg-Trp replacement in the mature protein (6). In 1999 Ozata, Ozdemir & Licinio identified a new adult female homozygous patient in this extended family (1). 1 boy with an identified leptin gene mutation will be studied under this protocol. We have recently identified and will recruit this patient with congenital leptin deficiency resulting in early-onset obesity.

12.7.2 What measures will be taken during the recruitment process to safeguard against the potential coercion or the appearance of coercion of participants, particularly vulnerable populations?

We will explain the protocol thoroughly in english or turkish.

**12.8** **Informed Consent:**

12.8.1 **Consent Process:** Please explain the process of obtaining consent from research participants. Under what setting and conditions will consent be obtained? What measures will be taken to ensure that subjects will make their decisions independently?

**Personnel Inviting Participants:**

Individuals authorized to solicit consent and sign the consent form confirming that the prospective subject was provided the necessary information and that any question asked was answered are enlisted below:

| **Name** | **Address** | **Daytime Phone** |
| --- | --- | --- |
| Julio Licinio, M.D. | UM/JMH Mental Health Hospital Center  1695 NW 9th Avenue, Suite 3100  Miami, Florida  33136 | (305)355-9105 |
| Ma-Li Wong M.D. |  |  |
| Luciana Ribeiro, M.D |  |  |
| Joao Vicente Busnello, MD |  |  |

**Process of Consent;**

The consent form and procedures will be explained to the mother via teleconference with the study investigators while the mother and the boy are still in Turkey. After all procedures are explained the mother will be asked to sign consent form.

**Comprehension of the Information Provided:**

The child's parent' comprehension about the research will be assessed by questioning her on the various items of the consent form. Since the mother speaks Turkish only, Dr. Yildiz, a co-investigator who is fluent in Turkish, will be mediating all communication. A Turkish translation of the consent form will also be provided.

**Information Withheld From Subjects:**

No information about the research purpose and design will be withheld

**Consent / Assent Forms:**

Consent form will be used in a English and Turkish formats

- - 1. **Capacity to Consent:** Complete this question only if enrolling participants with limited decision-making capacity. How will capacity to consent be assessed? Who will give this assessment?

The subject legally authorized representative will sign the informed consent form.

**REFERENCES:**

1. Troiano RP, Flegal KM. Overweight children and adolescents: description, epidemiology, and demographics. Pediatrics 1998;101(3 Pt 2):497-504.

2. Dietz WH. Health consequences of obesity in youth: childhood predictors of adult disease. Pediatrics 1998;101(3 Pt 2):518-25.

3. Zhang Y, Proenca R, Maffei M, Barone M, Leopold L, Friedman JM. Positional cloning of the mouse obese gene and its human homologue. Nature 1994;372(6505):425-32.

4. Prolo P, Wong ML, Licinio J. Leptin. Int J Biochem Cell Biol 1998;30(12):1285-90.

5. Licinio J, Negrao AB, Mantzoros C, Kaklamani V, Wong ML, Bongiorno PB, et al. Sex differences in circulating human leptin pulse amplitude: clinical implications. J Clin Endocrinol Metab 1998;83(11):4140-7.

6. Himms-Hagen J. Physiological roles of the leptin endocrine system: differences between mice and humans. Crit Rev Clin Lab Sci 1999;36(6):575-655.

7. Montague CT, Farooqi IS, Whitehead JP, Soos MA, Rau H, Wareham NJ, et al. Congenital leptin deficiency is associated with severe early-onset obesity in humans. Nature 1997;387(6636):903-8.

8. Farooqi IS, Jebb SA, Langmack G, Lawrence E, Cheetham CH, Prentice AM, et al. Effects of recombinant leptin therapy in a child with congenital leptin deficiency. N Engl J Med 1999;341(12):879-84.

9. Strobel A, Issad T, Camoin L, Ozata M, Strosberg AD. A leptin missense mutation associated with hypogonadism and morbid obesity. Nat Genet 1998;18(3):213-5.

10. Ozata M, Ozdemir IC, Licinio J. Human leptin deficiency caused by a missense mutation: multiple endocrine defects, decreased sympathetic tone, and immune system dysfunction indicate new targets for leptin action, greater central than peripheral resistance to the effects of leptin, and spontaneous correction of leptin-mediated defects. J Clin Endocrinol Metab 1999;84(10):3686-95.

11. Licinio J, Negrao AB, Mantzoros C, Kaklamani V, Wong ML, Bongiorno PB, et al. Synchronicity of frequently sampled, 24-h concentrations of circulating leptin, luteinizing hormone, and estradiol in healthy women. Proc Natl Acad Sci U S A 1998;95(5):2541-6.

12. Fantuzzi G, Faggioni R. Leptin in the regulation of immunity, inflammation, and hematopoiesis. J Leukoc Biol 2000;68(4):437-46.

13. Ducy P, Amling M, Takeda S, Priemel M, Schilling AF, Beil FT, et al. Leptin inhibits bone formation through a hypothalamic relay: a central control of bone mass. Cell 2000;100(2):197-207.

14. Farooqi IS, Matarese G, Lord GM, Keogh JM, Lawrence E, Agwu C, et al. Beneficial effects of leptin on obesity, T cell hyporesponsiveness, and neuroendocrine/metabolic dysfunction of human congenital leptin deficiency. J Clin Invest 2002;110(8):1093-103.

15. Baroncelli GI, Saggese G. Critical ages and stages of puberty in the accumulation of spinal and femoral bone mass: the validity of bone mass measurements. Horm Res 2000;54 Suppl 1:2-8.

16. Carter DR, Bouxsein ML, Marcus R. New approaches for interpreting projected bone densitometry data. J Bone Miner Res 1992;7(2):137-45.

17. Katzman DK, Bachrach LK, Carter DR, Marcus R. Clinical and anthropometric correlates of bone mineral acquisition in healthy adolescent girls. J Clin Endocrinol Metab 1991;73(6):1332-9.

18. Jergas M, Breitenseher M, Gluer CC, Yu W, Genant HK. Estimates of volumetric bone density from projectional measurements improve the discriminatory capability of dual X-ray absorptiometry. J Bone Miner Res 1995;10(7):1101-10.

19. Kroger H, Kotaniemi A, Vainio P, Alhava E. Bone densitometry of the spine and femur in children by dual-energy x-ray absorptiometry. Bone Miner 1992;17(1):75-85.

20. Kroger H, Kotaniemi A, Kroger L, Alhava E. Development of bone mass and bone density of the spine and femoral neck--a prospective study of 65 children and adolescents. Bone Miner 1993;23(3):171-82.

21. Lu PW, Cowell CT, SA LL-J, Briody JN, Howman-Giles R. Volumetric bone mineral density in normal subjects, aged 5-27 years. J Clin Endocrinol Metab 1996;81(4):1586-90.

22. Peel NF, Eastell R. Diagnostic value of estimated volumetric bone mineral density of the lumbar spine in osteoporosis. J Bone Miner Res 1994;9(3):317-20.

23. Matkovic V, Jelic T, Wardlaw GM, Ilich JZ, Goel PK, Wright JK, et al. Timing of peak bone mass in Caucasian females and its implication for the prevention of osteoporosis. Inference from a cross-sectional model. J Clin Invest 1994;93(2):799-808.

24. Plotkin H, Nunez M, Alvarez Filgueira ML, Zanchetta JR. Lumbar spine bone density in Argentine children. Calcif Tissue Int 1996;58(3):144-9.

25. Warner JT, Cowan FJ, Dunstan FD, Evans WD, Webb DK, Gregory JW. Measured and predicted bone mineral content in healthy boys and girls aged 6-18 years: adjustment for body size and puberty. Acta Paediatr 1998;87(3):244-9.

26. Molgaard C, Thomsen BL, Prentice A, Cole TJ, Michaelsen KF. Whole body bone mineral content in healthy children and adolescents. Arch Dis Child 1997;76(1):9-15.

27. Prentice A, Parsons TJ, Cole TJ. Uncritical use of bone mineral density in absorptiometry may lead to size-related artifacts in the identification of bone mineral determinants. Am J Clin Nutr 1994;60(6):837-42.

28. Felson DT, Zhang Y, Hannan MT, Anderson JJ. Effects of weight and body mass index on bone mineral density in men and women: the Framingham study. J Bone Miner Res 1993;8(5):567-73.

29. Ruegsegger P, Elsasser U, Anliker M, Gnehm H, Kind H, Prader A. Quantification of bone mineralization using computed tomography. Radiology 1976;121(1):93-7.

30. Hangartner TN, Overton TR. Quantitative measurement of bone density using gamma-ray computed tomography. J Comput Assist Tomogr 1982;6(6):1156-62.

31. Gilsanz V, Kovanlikaya A, Costin G, Roe TF, Sayre J, Kaufman F. Differential effect of gender on the sizes of the bones in the axial and appendicular skeletons. J Clin Endocrinol Metab 1997;82(5):1603-7.

32. Steiger P, Block JE, Friedlander A, Genant HK. Precise determination of paraspinous musculature by quantitative CT. J Comput Assist Tomogr 1988;12(4):616-20.

33. Knapik JJ, Staab JS, Harman EA. Validity of an anthropometric estimate of thigh muscle cross-sectional area. Med Sci Sports Exerc 1996;28(12):1523-30.

34. Kalender WA. Effective dose values in bone mineral measurements by photon absorptiometry and computed tomography. Osteoporos Int 1992;2(2):82-7.

35. Keens TG, Oki GS, Gilsanz V, Roe TF, Goodman W, Tsang RC, et al. Does radiation research in healthy children pose greater than minimal risk? Irb 1994;16(5):5-10.

36. Goldman M. Risks from low doses of radiation: continued. Science 1996;273(5275):562-4.

37. Lewis MK, Blake GM, Fogelman I. Patient dose in dual x-ray absorptiometry. Osteoporos Int 1994;4(1):11-5.

38. Evans RA, Marel GM, Lancaster EK, Kos S, Evans M, Wong SY. Bone mass is low in relatives of osteoporotic patients. Ann Intern Med 1988;109(11):870-3.

39. Adler YT, Fernbach SK, Hayman LA, Redman HC, Rumack C. The impact of maternity on radiologists: the AAWR position and its acceptance by women. AJR Am J Roentgenol 1986;146(2):415-7.

**Appendix 1: Study Drug Administration**

Injections at a single site will have a maximum allowable volume of 2.0 ml. Study medication should be administered preferably at the same time each day. The site(s) of injection should not occur on the limb from which subsequent blood draws will occur that day.

***Guide to Injection***

Setting Up

Inject preferably at the same time each day.

Find a comfortable, well-lighted work area for injection.

Clean the work area, preferably with alcohol.

Wash hands, preferably with liquid soap.

Remove a vial of study drug from the refrigerator and let it reach room temperature (5 minutes). (Do not shake or agitate the vial.)

Assemble these supplies in the selected work area:

Vial(s) of lyophilized study drug

Vial(s) of sterile water for injection

Latex-free syringes and needles (1 syringe/needle for reconstitution and 1 syringe/needle for self-administration per vial)

Alcohol swabs

Gauze

Band-Aid

Sharps container

Reconstitute the study drug as directed.

***Selection & Preparation of the Injection Site***

Step 1. Holding the syringe of study drug in one hand, select one of the injection sites shown in diagrams 1 and 2 using the other hand. *(Be sure to alternate injection sites each time, to avoid soreness at any site.)*


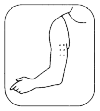

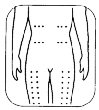


**Diagram 1 Diagram 2**

Step 2. Clean the site with an alcohol swab, using circular motions.

Step 3. Insert the needle straight into the injection site at a 45-degree angle.

Step 4. Pull back on the plunger slightly, if blood appears in the syringe, remove the syringe and choose a different site. Repeat steps 2, 3 and 4 at the new site.

Step 5. If there is no blood in the syringe, inject all of the study drug by pushing the plunger all the way down.

Step 6. After injecting the study drug, remove the needle from the skin and place an alcohol swab over the injection site.

Step 7. Discard the needle in the sharps container.

*Injecting the Dose*


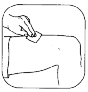

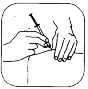

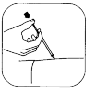

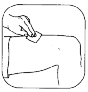


**Diagram 3 Diagram 4 Diagram 5 Diagram 6**

Step 2 Steps 3 & 4 Step 5 Step 6
